# Salivary cortisol and alpha-amylase as stress markers to evaluate an individualized music intervention for people with dementia: feasibility and pilot analyses

**DOI:** 10.1186/s13104-024-06904-7

**Published:** 2024-09-12

**Authors:** Mareike Christina Hillebrand, Cornelia Sindermann, Christian Montag, Alexandra Wuttke, Rebecca Heinzelmann, Heidrun Haas, Gabriele Wilz

**Affiliations:** 1 Department for Counseling and Clinical Intervention, Friedrich-Schiller-University Jena, Jena, Germany; 2 Institute of Psychology and Education, Ulm University, Ulm, Germany; 3 Computational Digital Psychology, Interchange Forum for Reflecting on Intelligent Systems, University of Stuttgart, Stuttgart, Germany; 4 Center for Mental Health, Würzburg University Hospital, Würzburg, Germany; 5 Department of Psychiatry and Psychotherapy, University Medical Center, Johannes Gutenberg-University Mainz, Mainz, Germany

**Keywords:** Cognitive impairment, Psychobiology of stress, ANS, HPA axis, Non-pharmacological intervention, Cortisol, Alpha-amylase, Salimetric’s children’s Swab

## Abstract

**Objectives:**

We investigated salivary biomarkers of stress, more specifically, cortisol and alpha-amylase, to evaluate effects of individualized music listening (IML) in people with dementia.

**Method:**

Participants were *N* = 64 nursing home residents with dementia (mean_age_ = 83.53 ± 7.71 years, 68.8% female). Participants were randomly assigned to either listening to their favorite music every other day for a period of six weeks (intervention), or standard care (control). Using the Saliva Children`s Swab (SCS), saliva was collected before, after, and 20 min after IML sessions at the beginning and end of the intervention period for the analysis of salivary alpha-amylase and cortisol.

**Results:**

Using the SCS was feasible in people with dementia. Nevertheless, there was no effect of IML on salivary stress markers.

**Discussion:**

Although using SCS was feasible, active patient engagement is required. Future studies need to corroborate findings in larger samples.

**Trial registration:**

German Clinical Trials Register: DRKS00015641, ISRCTN registry: ISRCTN59052178.

**Supplementary Information:**

The online version contains supplementary material available at 10.1186/s13104-024-06904-7.

## Introduction

The burdensome behavioral and psychological symptoms of dementia (BPSD), such as agitation, depression, or apathy, are said to be related to the dysregulation of physiological stress systems [1]. According to Gerdner’s mid-range theory [2], individualized music listening (IML) can be seen as an interpretable stimulus that is associated with positive feelings and thus leads to relaxation, expressed by reduced BPSD. However, investigations on endocrine, autonomic and immune responses to IML interventions are hardly existing. In a recent literature review [3], it was found that only three studies investigated effects of IML using biomarkers of stress, with preliminary evidence, at least in terms of autonomic activity, for a biological stress-reduction after IML. A recent paper found also a positive indication for reduced salivary cortisol (sCort), as an indicator of the hypothalamic–pituitary–adrenal (HPA) axis activity [4].

Nonetheless, more evidence on effects of IML on biological stress marker would be important also because of the difficulties in assessing subjective stress in people with dementia, especially in advanced stages of dementia [5]. The assessment of biomarkers of stress can be considered as objective measure and does not rely on the introspection abilities of individuals [6]. In consequence, it represents one of few remaining methods to capture the perspective of the people with dementia.

Cortisol is an already well-established biomarker in research investigating stress in people with dementia [7]. In contrast, salivary biomarkers of the ANS are less extensively investigated in this population, even if salivary alpha-amylase (sAA) has been already shown to be a sensitive stress measure for the evaluation of non-pharmacological interventions [8]. There are also promising results on sCort and sAA in relation to stress-reducing effects of music listening in healthy participants [9, 10] and caregivers [11].

However, several challenges must be considered when measuring saliva in this population [12–14], but little is known about whether and how to collect/analyze salivary biomarkers in people with (especially advanced) dementia.

Thus, the aim of the present study was to test whether saliva sampling is feasible in people with dementia in every stage of severity (i.e., acceptability), and whether the quality and volume of saliva samples is sufficient for testing. We also aimed to analyze the effects of IML on biomarkers of stress in people with dementia.

## Method

### Study design and setting

This study was conducted as a side project of a Randomized Controlled Trial (RCT) that aimed to evaluate an IML intervention for people diagnosed with dementia in institutional care. Data was collected between 2018 and 2020 in German nursing homes. Details on the intervention and the study design are provided in the study protocol [15] and the supplemental material. Ethical approval was obtained from the Ethics Committee of the Faculty of Social and Behavioral Sciences of the Friedrich Schiller University Jena (committee’s reference number: FSV 18/39 & 19/09). The person with dementia or legally authorized representatives gave written informed consent prior to participation. The trial was registered in the German Clinical Trials Register (DRKS00015641) and ISRCTN registry (ISRCTN59052178). We aimed for the study to adhere to CONSORT guidelines.

### Saliva collection protocol

Data were assessed at the beginning (T1) and the end (T2) of six weeks of IML intervention. Saliva samples were collected at each of these two assessment points at three time points: directly before the IML session started, directly after the end of the IML session and 20 min after the end of the IML session. The control group provided saliva in similar time frames (for details on the saliva collection protocol see the supplemental material).

### Measures

Sociodemographic data, cognitive impairment, and agitation were included in the analyses of the feasibility of saliva collection as control variables. These data were assessed via questionnaires answered by the nursing staff. Details on measures used here, i.e., the Mini-Mental-State Examination [16] and the Cohen Mansfield Agitation Inventory [17], can be found in the study protocol [15].

### Saliva collection method

We used a method that requires a low level of understanding and was already evaluated successfully for small children [18]: the SalivaBio Children’s Swab (SCS; Salimetrics, PA; Item no. 5001.06; results on a pilot trial using passive drool method are reported in in the supplemental material as well as details on saliva handling and processing).

### Statistical analyses

The feasibility analyses were conducted in SPSS (Version 28.0, IBM Corp.) in addition to the effectiveness analyses. Specifically, binary variables on the success of saliva collection/ successful testing with sufficient volume were correlated with agitation, cognitive impairment and medication using the correlation method that was appropriate for the metrics of the specific variables. Analyses on the effectiveness of the intervention were conducted in JASP (Version 0.16) by calculating Bayesian repeated measures ANOVA with sCort and sAA as our primary outcome. The detailed analysis plan is reported in the supplemental material.

## Results

**Fig. 1:**
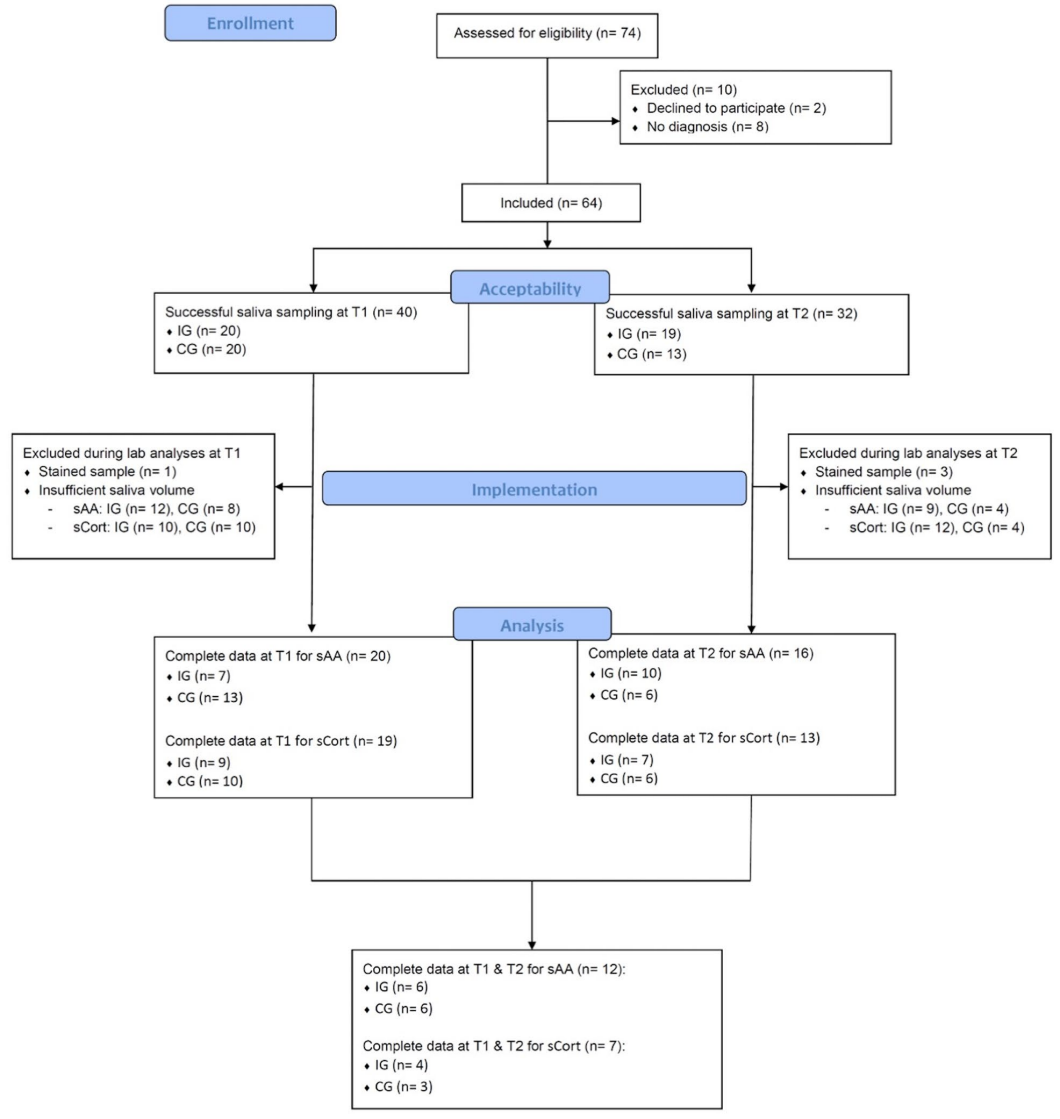
Flow diagram displaying the steps of enrollment, saliva collection and analyses

The sample characteristics are displayed in Table 1. Detailed information on the flow of participants is displayed in Fig. 1 regarding successful collected/analyzed saliva samples per participant.

**Table 1:** Sociodemographic description of both treatment groups (*n*_*IG*_ = 31, *n*_*CG*_ = 33)

	IG			CG	
	*N*	(*N* in %)		*N*	(*N* in %)
Female	23	(74.2%)		21	(63.6%)
Disorder					
Alzheimer’s Disease	6	(19.4%)		16	(48.5%)
Vascular dementia	2	(06.5%)		1	(03.0%)
Unspecified	7	(22.6%)		3	(09.1%)
Dementia in other diseases	16	(51.6%)		13	(39.4%)
Stage of severity (MMSE)					
Mild	5	(16.1%)		3	(09.1%)
Moderate	9	(29.0%)		6	(18.2%)
Severe	17	(54.8%)		24	(72.7%)
Psychopharmacological treatment	25	(80.7%)		25	(75.8%)
Treatment with cortisone	0	(00.0%)		0	(00.0%)
Treatment with beta-blocker	15	(48.4%)		9	(27.3%)
	*M*	*SD*		*M*	*SD*
Age	83.00	08.41		84.03	07.09
MMSE score	08.10	01.50		07.00	01.23
Mild	20.40	0.68		22.33	2.40
Moderate	14.22	1.10		12.33	1.31
Severe	1.24	0.55		3.29	0.75

Note. Intervention group (IG), control group (CG), M and SD represent the mean and standard deviation. Stage of severity was assessed with the German version of the Mini-Mental State examination (MMSE, Folstein et al., 1975). The severity of the stages was defined as follows: mild: ≥19, moderate: 10–18, severe: ≤9

### Saliva collection

Out of the *N* = 64 participants, who agreed to participate in this study, *N* = 40 participants (62.5%) accepted the swab method at T1 and *N* = 32 participants (62.7%) at T2. Across the treatment groups, it was only possible to apply the method on *N* = 25 participants at T1 *and* T2 (39.1%, *n*_*IG*_ = 10, *n*_*CG*_ = 15, 76% female, *M*_*age*_ = 83.48 y., *SD*_*age*_ = 6.55 y., *M*_*MMSE*_ = 10.84, *SD*_*MMSE*_ = 8.21, 24% were mildly, 32% moderately, and 44% were severely cognitively impaired). Reasons for drop-out were incomprehension (31.28%), (non-)verbal refusal (24.24%), agitation (24.24%), dry mouth (8.88%), discomfort/weakness (8.88%) and aggression (2.22%). Cognitive impairment was significantly correlated with the success of method application at T1 (*r*(62) = 0.47, *p* < .001, see Table S1 in the supplemental material).

### Successful testing

The analysis of sCort was only successful for all people with dementia, who provided sufficient and blood-free saliva volume (at least 100µL per sample (in duplicate): *n*_*T1*_ = 19 participants (47.5%), *n*_*T2*_ = 13 participants (40.63%)). Only *n* = 7 participants provided enough saliva for analyses at both time points (T1/T2). The average intra-assay coefficient of variation (CV) was 5.05% and the average inter-assay CV was 6.30%.

Using a kinetic measurement of sAA activity, a satisfying intra-assay CV (1.80%) and inter-assay CV was found (3.36%). The volume of saliva required was 10 µL plus some handling volume per sample. Valid samples were finally obtained by *n =* 20 participants at T1 (50%) and *n =* 16 participants at T2 (50%). Only *n* = 12 participants provided valid saliva samples for sAA at both time points. Sufficient salivary volume for both biomarkers was not related to the medications or the severity of dementia (see Table S2 in the supplemental material).

### Effectiveness

**Fig. 2:**
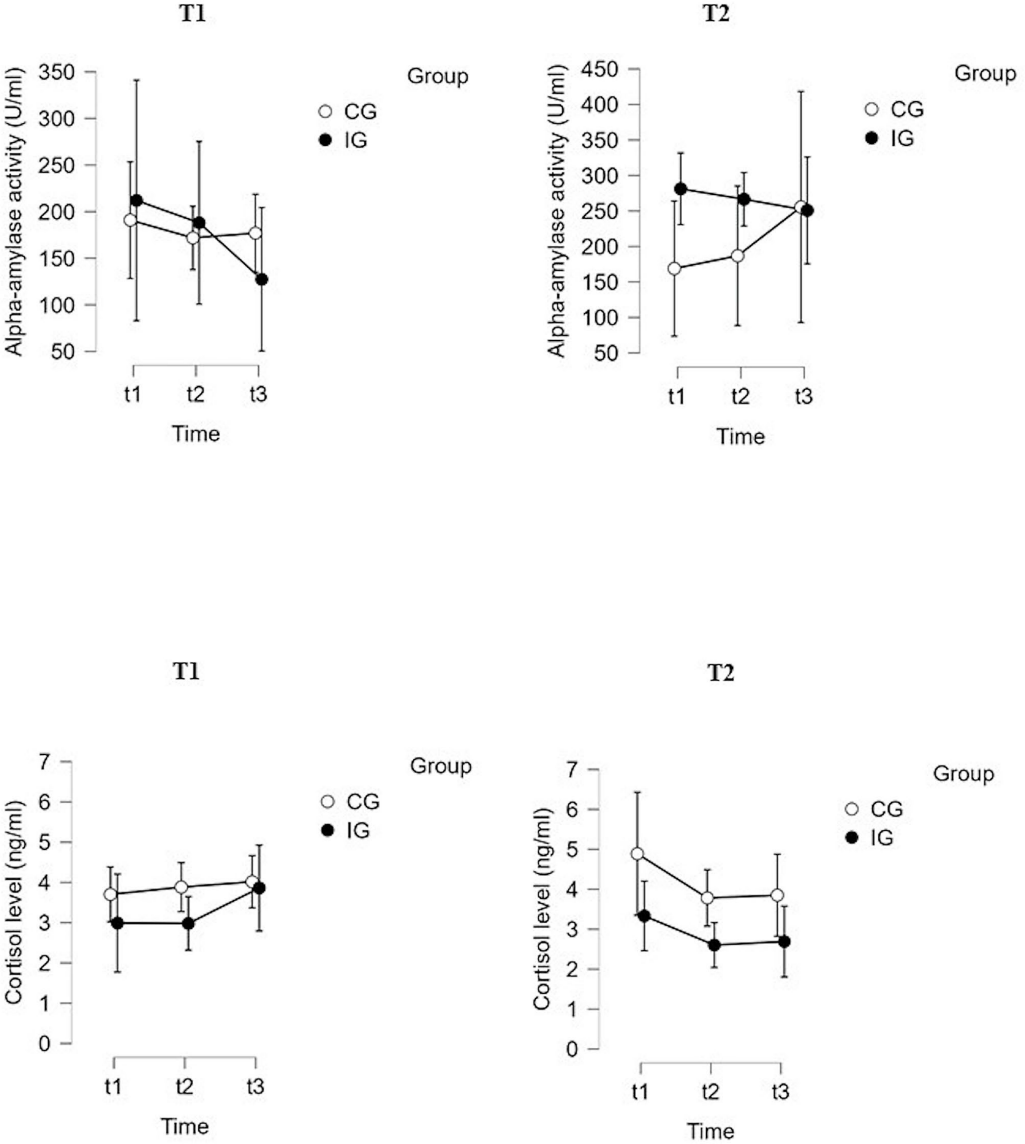
Salivary alpha-amylase activity (U/ml) and cortisol levels (ng/ml) in the control group (CG) and intervention group (IG) at the three assessment points (t1: before music listening, t2: after music listening, t3: 20 min after music listening) at the beginning (T1) and the end (T2) of 6 weeks of intervention period

The plots in Fig. 2 show a systematic trend for sAA over time. Across the three measurements at both T1 and T2, sAA activity decreased in the IG, but not in the CG. For sCort secretion, the plots are not clearly interpretable in terms of possible trends (see Fig. 2). To measure effects of IML BF_incl_ were calculated and interpreted (see Table S3 in the supplemental material). Since BF_incl_ were predominantly between 0.1 and 0.7, evidence for the absence of an effect of IML on salivary stress markers was given.

## Discussion

The aim of the present study was not only to investigate whether saliva sampling and testing is feasible in people with dementia in every stage of the illness, but also to analyze effects of IML on biomarkers of stress. During data collection for this study, challenges in collecting saliva samples became apparent. Thus, the feasibility of saliva measures in people with (advanced) dementia was in question. From previously *N* = 74 enrolled participants, only *n* = 12 participants provided valid saliva samples for sAA at the beginning and the end of the intervention period. The number of participants was even smaller for sCort (*n* = 7 participants). Therefore, only exploratory analyses on the physiological effects of IML could be performed, and the results must be interpreted with caution.

Considering saliva collection, using the SCS allowed us to collect all saliva samples at least at one of the two assessment days from 62.5% of the participants. It should be noted, that cognitive impairment was positively associated with successful application of the SCS at the beginning of the intervention. Respectively, higher cognitive impairment was associated with unsuccessful saliva collection. Nevertheless, this association was not found at T2, leading to the assumption that the SCS may still be feasible in advanced stages of dementia, but high drop-out quotes must be considered.

A main challenge in samples of people with dementia is also the insufficient saliva flow due to hyposalivation or xerostomia [19]. Multiple medications pose a high risk to reduced saliva flow [20]. Even if in the present sample no association was found between sufficient saliva volume and medication, a possible interaction cannot be excluded, since 78.1% participants took psychopharmacological medication regularly.

Beside studies reporting the same challenges while saliva collection and analyses as described above [13], there are also studies reporting small drop-out quotes in their samples, even if they also predominantly assessed people with advanced dementia. These studies primarily used other collection devices as the Salivette™ [21]. In contrast, the SCS was used in another study that found immense drop-out quotes [13]. The difference between the Salivette ^TM^ and the SCS is that the Salivette ^TM^ allows chewing on the material during collection. Since chewing can stimulate saliva secretion [22], the different drop-out quotes regarding sufficient saliva volume can be a result of the choice of the collection device and the way of collection.

Beside the above-mentioned challenges in data collection, preliminary data on physiological stress reduction following IML in people with dementia was obtained and interpreted. The descriptive findings in the present RCT suggest stress-reducing effects after IML, when considering the ANS activity, even when the promising trends of sAA activity can only be interpreted cautiously due to non-significant findings and small sample size. It is nevertheless interesting that the descriptive trends found for sAA activity can be interpreted in line with findings from previous studies suggesting an autonomic down-regulations after IML in people with dementia [3]. However, effects on endocrine functioning are still unknown, since even the descriptive plot analyses for sCort revealed no systematic trend, as it was found for sAA activity. The finding of missing systematic trends for sCort is in line with previous studies that reported conflicting findings on effects of music-based interventions (including music therapy) on the functioning of the HPA axis, with some studies indicating reduced sCort levels after music-based interventions and some studies reporting no effects [3, 4, 11].

### Limitations

The number of the final sample was decreased to *N* = 12 participants in comparison to the previously planned *N* = 50 participants. Thus, statistical power may be reduced. We used Bayes analyses, as this statistical method is less sensitive to power restrictions [23]. Nevertheless, a lack of statistical evidence may be due to the small sample size or uncontrolled third variables (e.g., medication, severity of dementia). Future research should consider potential third variables in sufficiently powered trials that also assess distinctive diurnal patterns of both biomarkers [21, 24]. It is also not known whether IML works better than (active) music therapy [25, 26]. Future research should compare IML with active music therapy to make informed treatment recommendations.

## Conclusion

Despite these limitations, this RCT revealed that (i) the collection of saliva is in general feasible using the SCS, however (ii) the volume of saliva was often insufficient for the determination of even one of the two biomarkers. Finally, (iii) sAA activity may be a sensitive biomarker for stress-reducing effects of IML in people with dementia but using this biomarker needs high expertise in the execution of assays. In summary, it can be concluded that salivary measures can be used to investigate physiological mechanism underlying potential positive effects of non-pharmacological interventions for people with dementia, but multiple challenges must be considered.

## Electronic supplementary material

Below is the link to the electronic supplementary material.

Supplementary Material 1

## Data Availability

The data that support the findings of this study are available from the corresponding author upon reasonable scholarly request.

## Abbreviations

ANS: Autonomous Nervous System
BPSD: Behavioral and Psychological Symptoms of Dementia
HPA axis: Hypothalamic–pituitary–adrenal axis
IML: Individualized Music Listening
RCT: Randomized Controlled Trial
sAA: Salivary Alpha-Amylase
sCort: Salivary Cortisol
SCS: Salimetric’s Children Swab
