# Supplementary material for: Salivary cortisol and alpha-amylase as stress markers to evaluate an individualized music intervention for people with dementia: feasibility and pilot analyses
Source: BMC Res Notes. 2024 Sep 12;17:258. doi: 10.1186/s13104-024-06904-7 (PMC11391792; doi:10.1186/s13104-024-06904-7)
Supplement: Supplementary file 1 — Supplementary Material 1 [file 13104_2024_6904_MOESM1_ESM.pdf]

Intervention protocol

Individualized music listening sessions were provided via mp3-player and headphones every other day for 20 minutes over a period of six weeks. The project members created during the intervention period up to three playlists based on information provided by relatives, nursing home staff and of course from the participants themselves, if possible. Playlists were continuously adapted over the intervention period as needed. Furthermore, music listening was monitored by the project staff or nursing home staff. Participants allocated to the control group received standard care. Participants of both groups participated in simultaneous offers in the nursing homes.

Sample size and randomization

This study was performed as a side project of a trial investigating the effectiveness of an individualized music intervention for people with dementia in institutional care (German Clinical Trials Register: DRKS00013793; ISRCTN registry: ISRCTN59052178). Thus, sample size calculations were based on the specifications of the main project and by reaching the predefined sample size of this main study (see the study protocol for details), recruitment stopped also for this side study. Randomized allocation was performed in a 1:1 ratio stratified by gender using a computer-generated randomization list for each nursing home. The randomization was conducted by a member of the working group, who was not involved in the baseline assessment using the random number generator Random.org.

Results from a pilot trial on the feasibility of passive drool method

Before the beginning of the present RCT, we conducted a small pilot study with N = 4 participants randomly selected from the main sample (n_{IG} = 2 participants, n_{CG} = 2 participants) to find an appropriate saliva collection method for people with dementia. We investigated the feasibility of the passive drool (PD) method, which requires participants to not swallow for one to two minutes and transfer all accumulated saliva through a straw into a specific collection tube afterwards. None of the participants were we able to collect any saliva

using this method due to incomprehension of the instruction (*n* = 1), lack of consent (*n* = 1), physical weakness (*n* = 1), and agitation (*n* =1).

Saliva collection using the Salimetric's Children Swab (SCS)

After the pilot test of the feasibility of the PD method described above, we decided to use Salimetric's Children Swab (SCS, exclusively from Salimetrics, State College, PA) in the main study. The SCS is an alternative to the commonly used Salivette (Salivettes®, SARSTEDT, Numbrecht, Germany) as it is designed for children to facilitate saliva collection. The use of Salivettes requires understanding and correctly performing a series of steps that can be as confusing for children and people with dementia as the PD method (see also Granger et al., 2007 or Fey at al., 2024 for an overview). In addition, the SCS reduces the potential risk of swallowing the swab (i.e., the SCS is a 125mm swab that can be held at one end while the other end is placed in the mouth of the person with dementia, eliminating the risk of swallowing). We therefore decided to use the SCS because it is easy and less stressful to handle.

Saliva collection was in general conducted in the afternoon between app. 2:30 and 5:30 p.m. The duration of taking samples and reasons for failures of attempts to collect saliva samples were protocolled. Physically exhaustive activities two hours prior to/during the assessments, the consumption of caffeine, nicotine or alcohol within two hours before/during the assessment, and chemotherapeutic treatment during the past year were recorded as possible confounding variables. Medication was recorded from the medical records of the participants. Saliva collection was carried out by trained (student) researchers.

Specifically, the SCS was placed under the participant's tongue for at least 2 minutes. Afterwards, it was put in Salimetrics storage tubes and immediately stored at 4℃ for max. 14 days. Within the 14 days, the samples were transported refrigerated at 4℃ to the laboratory at Ulm University, Germany. There the samples were centrifuged for 10 minutes at 2500 relative centrifugal force (rcf) and stored at -20℃ until further analysis. In the laboratory, the procedure of enzyme-linked immunosorbent assay (ELISA) was performed in duplicate to determine cortisol levels (Cortisol ELSA Kit, Item no. DES6611, demeditec, Germany) and

alpha-amylase levels (Alpha-Amylase ELSA Kit, Item no. DEEQ6231, demeditec, Germany). However, first results of the analyses of alpha-amylase levels did not hold up to prespecified quality. We therefore commissioned a laboratory (specifically daacro`s saliva lab, Trier, Germany) specialized in sAA analyses using a kinetic assay (α-Amylase Kinetic Enzyme Assay Kit, Item no. 1-1902, Salimetrics, USA) for the determination of sAA activity, which means that we changed the method and quality of sAA determination by assessing sAA activity rather that sAA levels.

Determination of salivary alpha-amylase levels

To determine alpha-amylase levels in the saliva samples, the procedure described in the booklet of the ELISA Kit was followed. However, after analyzing test samples (the laboratory in Ulm used this specific ELISA kit for the first time and, thus, started with test samples), results indicated some issues. More specifically, it became apparent that the coefficient of variance (CV) oftentimes exceeded 5% (more specifically, it lied between 35% and 57% several times), reflected in large differences in measured alpha-amylase levels between duplicate samples, controls, and standards. The aim to achieve a CV of around 5% or lower was based on specifications in the booklet of the ELISA Kit. In line with the high CV, also the standard curve to determine alpha-amylase levels of samples was not satisfactory. Additionally, many (>50%) of the measured alpha-amylase levels of controls were not in the range predefined in the kit.

To overcome these issues and in close collaboration with Demeditec, the procedure was closely monitored, and several improvements were implemented in the procedure. Not only was the time needed for pipetting decreased (e.g., by preparing plates with pre-diluted samples/controls/standards beforehand to enable the use of multichannel pipettes) but also the accuracy and precision of all pipetting steps was closely monitored and ensured (e.g., standard procedures were closely monitored and ensured: each pipetting step was monitored by two individuals to ensure precision and that none of the wells was damaged, new tips were used for each step of the procedure, all tips were wetted before the actual pipetting; moreover, pipettes were tested for precision before being used, and electrical

pipettes were used in one run). Additionally, it was tested whether more rigorous washing steps during the ELISA procedure would improve results (e.g., via diligently checking for bubbles in the wells which might cause issues during measurement, thorough washing steps, using new tips for every washing step to prevent contamination of wells, adding an additional washing step). All samples, standards, and controls were rigorously homogenized before the analyses, analyses were repeated with triplets instead of duplicates, and different kits from different batches were used in repeated analyses, as well. All these monitoring steps and improvements, however, did not help to completely overcome the aforementioned issues in the results. Putatively, a multitude of factors and their interactions contributed to the non-satisfactory results found in the context of the present study. Among others, the specific kits might not have been the best choice for the analyses of the present study. As mentioned by Demeditec, their alpha-amylase ELISA kits are a test system for in vitro determination of alpha-amylase in human saliva for diagnosis of diseases associated with *over*production of alpha-amylase, such as psychological and physical stress, pain syndrome (fibromyalgia), and for course and therapy control of stress disorder. Additionally, and in line with this, the present samples' alpha-amylase levels might have been outside the ideal range of concentration to be accurately measured by the specific kits used. In the end, however, we cannot conclude with certainty why the analyses did not work satisfactorily.

Analysis plan

To answer the research questions on the feasibility of saliva sampling, we analyzed the sum of participants who adhered to the saliva sample collection protocol. Furthermore, we evaluated the number of people with dementia that provided valid samples for ELISA. In addition, the success of the receipt of valid saliva samples was analyzed in relation to medication, agitated behaviors and cognitive impairment. This was done using the correlation methods that are appropriate for the metrics of the specific variables (if two dichotomous variables: φ-coefficient; if one dichotomous variable & one interval scaled variable: point biserial correlation coefficient). The Holm-Bonferroni method was used to

adjust for multiple testing. In chi-square statistics, the Monte Carlo approximation was used when cells had expected frequencies of less than five.

Finally, the effectiveness of the individualized music intervention[1] was analyzed by calculating Bayesian repeated measures ANOVA. The Bayesian approach was used, since the Bayes factors (BF) are interpretable regardless of sample size (Jarosz & Wiley, 2014). The inclusion Bayes factor (BF_{incl}) was calculated to assess the evidence for or against certain predictors across several models. Usually, $BF_{incl} < 1.0$ are considered as evidence against a (main/interaction) effect of the independent variable (group, time) on the dependent variable (sAA, sCort). $BF_{incl} > 1.0$ give evidence for the effect on the dependent variable. The default JASP prior for fixed effects were used (r scale prior width = 0.5, i.e., the range of possible effect sizes). The analyses were also conducted with a wider (r = 1) and narrower scale (r = 0.2) to assess the robustness of the analyses to the default uninformed prior.

References

Fey, J.M.H., Bikker, F.J. & Hesse, D. (2024). Saliva Collection Methods Among Children and Adolescents: A Scoping Review. *Molecular Diagnosis & Therapy, 28,* 15–26. https://doi.org/10.1007/s40291-023-00684-9

Granger, D. A., Kivlighan, K. T., Fortunato, C., Harmon, A. G., Hibel, L. C., Schwartz, E. B., & Whembolua, G. L. (2007). Integration of salivary biomarkers into developmental and behaviorally-oriented research: problems and solutions for collecting specimens. *Physiology & Behavior, 92*(4), 583-590. https://doi.org/10.1016/j.physbeh.2007.05.004

Jarosz, A. F., & Wiley, J. (2014). What are the odds? A practical guide to computing and reporting Bayes factors. *The Journal of Problem Solving, 7*(1), 2.

[1] Please note that at the beginning of this study, it was planned to also assess subjective stress, using a smiley assessment scale, as a secondary outcome measure, but most participants were not able to rate their subjective stress experience.

Tables

Table S1. *Point biserial correlation of agitation and cognitive impairment at baseline with the success of method application (0 = unsuccessful, 1 = successful) at the first (T1) and last (T2) behavioral observation.*

		N	*r*	p_{adj}
CMAI sum score	T1	59	-0.25	.174
	T2	48	-0.12	.406
MMSE score	T1	64	0.47	.000***
	T2	51	0.22	.236

Note. CMAI: Cohen-Mansfield Agitation Inventory. MMSE: Mini Mental State Examination with higher values indicating less cognitive impairment. Successful method application (0 = no, 1 = yes). * < .05, ** < .01, ***< .001. *p*-values were adjusted for multiple tests using Holm-Bonferroni method.

Table S2. *Correlation coefficients for medication (0 = no, 1 = yes) and cognitive impairment (MMSE score) at baseline with sufficient saliva volume (0 = no, 1 = yes) at the first (T1) and last (T2) behavioral observation for salivary alpha-amylase (sAA) and salivary Cortisol (sCort).*

		r	φ	p^b
Medication	T1_sAA	--	-0.108	.685[a]
	T2_sAA	--	0.101	.705[a]
	T1_sCort	--	-0.201	.392[a]
	T2_sCort	--	0.271	.235[a]
MMSE score	T1_sAA	0.12	--	.490
	T2_sAA	0.07	--	.691
	T1_sCort	0.19	--	.299
	T2_sCort	-0.24	--	.190

Note. MMSE: Mini Mental State Examination with higher values indicating less cognitive impairment. If two dichotomous variables: φ- coefficient. If one dichotomous variable & one interval scaled variable: point biserial correlation coefficient (r).[a]Monte Carlo p-values were calculated since one cell had less than 5 respondents. [b]When adjusted for multiple tests using Holm-Bonferroni method, all *p*-values were 1.00.

Table S3. *Bayes factors for inclusion (BF_{Incl}) of main effects and interactions with a Cauchy prior width of 0.2.*

	**BF_{Incl}**			
Effects	sAA_T1	sAA_T2	sCort_T1	sCort_T2
N	20	16	19	13
Time	0.261	0.170	0.419	1.856
Group	0.499	0.673	0.605	1.434
Time*Group	0.365	0.609	0.107	0.304

Note. Compares models that contain the effect to equivalent models stripped of the effect. Higher-order interactions are excluded. Analysis suggested by Sebastiaan Mathôd. Group (IG vs. CG). Time: saliva sampling before (t1), after (t2) and 20 minutes after music listening (t3). T1: at the beginning of 6 weeks of intervention period. T2: At the end of 6 weeks of intervention period.
